# The present and future of peer review: Ideas, interventions, and evidence

**DOI:** 10.1073/pnas.2401232121

**Published:** 2025-01-27

**Authors:** Balazs Aczel, Ann-Sophie Barwich, Amanda B. Diekman, Ayelet Fishbach, Robert L. Goldstone, Pablo Gomez, Odd Erik Gundersen, Paul T. von Hippel, Alex O. Holcombe, Stephan Lewandowsky, Nazbanou Nozari, Franco Pestilli, John P. A. Ioannidis

**Affiliations:** ^a^Department of Affective Psychology, Institute of Psychology, Eotvos Lorand University, Budapest 1063, Hungary; ^b^Department of History and Philosophy of Science and Medicine, Indiana University, Bloomington, IN 47405; ^c^Cognitive Science Program, Indiana University, Bloomington, IN47405; ^d^Department of Psychological and Brain Sciences, Indiana University, Bloomington, IN 47405; ^e^Booth School of Business, University of Chicago, Chicago, IL 60637; ^f^Psychology Department, Skidmore College, Saratoga Springs, NY 12866; ^g^Department of Computer Science, Norwegian University of Science and Technology, Trondheim 7491, Norway; ^h^Aneo AI Research, Trondheim 7031, Norway; ^i^LBJ School of Public Affairs, University of Texas, Austin, TX 78712; ^j^School of Psychology, University of Sydney, Sydney NSW 2006, Australia; ^k^School of Psychological Science, University of Bristol, Bristol BS81TU, United Kingdom; ^l^Department of Psychology, University of Potsdam, Potsdam 14469, Germany; ^m^Department of Psychology, College of Liberal Arts, The University of Texas, Austin, TX 78712; ^n^Department of Neuroscience, College of Natural Sciences, The University of Texas, Austin, TX 78712; ^o^Meta-Research Innovation Center at Stanford, Stanford University, Stanford, CA 94305; ^p^Department of Medicine, Stanford University, Stanford, CA 94305; ^q^Department of Epidemiology and Population Health, Stanford University, Stanford, CA 94305; ^r^Department of Biomedical Data Science, Stanford, CA 94305

**Keywords:** peer review crisis, publication system, scientific community

## Abstract

What is wrong with the peer review system? Is peer review sustainable? Useful? What other models exist? These are central yet contentious questions in today’s academic discourse. This perspective critically discusses alternative models and revisions to the peer review system. The authors highlight possible changes to the peer review system, with the goal of fostering further dialog among the main stakeholders, including producers and consumers of scientific research. Neither our list of identified issues with the peer review system nor our discussed resolutions are complete. A point of agreement is that fair assessment and efficient change would require more comprehensive and rigorous data on the various aspects of the peer review system.

Peer review is intended to support scientific integrity, correct errors, and democratize decisions about publication and funding. Critical voices have raised concerns about scientific errors, unreliability, lack of transparency, and bias in peer review (1–3). Some reviewers indulge in a variety of self-serving behaviors, even obstructing the publication of articles unfavorable to their own research (4) and self-appropriating ideas from unpublished manuscripts accessed during peer review (4). Biases that favor or disfavor research topics, institutions, geographic origin, and demographic characteristics of authors or participants threaten the ideal of impartiality in peer review (1, 5). The reliability of peer review is low, as reviewers of the same work often disagree with each other’s assessments (6). The validity of peer review has also been cast into doubt by studies showing that major manuscript errors are often missed (7). Reviewers may lack expertise in a submission’s specific area of research, and reviewers with the requisite expertise often lack the time and incentives to conduct a thorough and careful review.

Moreover, peer review is slow, perhaps slower than the modern needs and capabilities of scientific communication. Papers typically take several months to be evaluated (6) and may take years to go through an entire cycle of rejection-revision that commonly occurs before publication. These long timelines compare unfavorably to the accelerating rate of scientific research (8), the instantaneous audience a paper can receive when posted on a preprint server, or the short timelines available for the business, policy, and treatment decisions which research is often meant to inform. Scientists seeking to share findings often encounter a peer review system that is slow, inefficient, costly, haphazard, biased, subject to abuse, and inaccurate (9).

Nevertheless, many scientists still see the utility in reviewing for various reasons—such as keeping up with their field, influencing the field’s direction, and (for junior scientists) learning how to evaluate research (10). Reviewed and reviewing scientists may become closer in their social network, even when reviewing is single- or double-blind (11), and contribute together to standardizing evaluative criteria in their field (12). More fundamentally, contributing reviews to one’s field is a central mechanism for creating a scientific community.

This perspective article is based on conversations among the authors and presentations at a recent meeting on scientific reform. Although we do not provide definitive solutions, our aim is to advocate for a reevaluation of the peer review process. To this end, we outline the problems and discuss the pros and cons of a menu of potential solutions. Our argument is outlined broadly in Table 1.

**Table 1.** The main problems with the current review system and some of their potential solutions

Category	Problems	Potential Solution	Advantages	Disadvantages
Quality	Lack of reviewers	Monetary compensation	More reward	Only wealthy publishers can afford it
		Preprint peer review	Large public reviewer pool Timely dissemination Less publication bias	Rare engagement of the public Misinformation from preprints Unclear impact of bias
		Improved recruitment	Various resources Crowdsourcing methods	Need validation of benefits
		AI-assisted reviews	Massive workforce	Questionable quality Requires human validation
	Lack of qualified reviewers	Reviewer training	Better preparation Standardization	Requires resources Standardization
	Insufficient scrutiny fueling irreproducibility	More reviewers Open and transparent reviews	More thorough review More information Better documentation	Slower process Variable implementation Hard to reject papers
	Increased specialization	Checklists (Ensure that important topics are covered)	Standardization	Unproven effectiveness
		Signed reviews (one can see which specialization was covered in the review)	Higher quality Fewer unprofessional comments Conflicts identified	Less critical reviews Fear of retaliation Actual retaliation
Predatory journals	No review or rudimentary review	Transparent editorial process + Signed reviews, Open and transparent reviews	Clarity how decisions are made	Uncertainty about best practices
Biases	Bias for or against authors, topics, methods, groups, institutions, countries, arguments, ideas	Double-blind peer review	Reduced biases	Possibly more critical reviews Often hard to achieve
		Publishing reviews + Signed reviews, Open and transparent reviews, Reviewer training	More transparency	Possibly less critical reviews
Poor reliability	Limited agreement and frequent disagreement between reviewers	Focus revisions on points where reviewers agree, and where they have special expertise	Higher reliability	Difficulty understanding what drives (dis)agreement between reviewers
	Lack of evidence for deciding how to improve the system	Empirical test and randomized trials	More rigorous evidence	Relatively few examples Difficult to implement Not easy to perform in real world

Listed advantages and disadvantages correspond to the solutions in bold.

## Problems with the Current Review System

### Quality Problems.

The quality of peer review has been questioned for quite some time. Early work highlighted reviewer biases—biases against new ideas and arguments (13) and biases in favor of well-known authority figures (14). Lately, issues related to the reproducibility crisis and increased scientific fraud have been partially attributed to poor-quality peer reviews that provide insufficient scrutiny (15). Metaresearch has found recurring issues in peer-reviewed articles. In psychological journals, for example, 18% of statistical results were found to be incorrectly reported (16), and the inconsistencies discovered in *P*-value reporting were demonstrated to affect conclusions once in every eight cases (17). Many studies highlight poor reporting of research outcomes and details of statistical analyses (18–21) and reported results being irreproducible from the data (22). These issues have led to an increasing rate of articles being retracted after publication (23) and suggest that reviews before publication often fail to catch important errors.

Perhaps the growth in the number of scientific publications and the increased specialization of scientific knowledge is causing a decline in the quality of peer review. It is difficult to recruit reviewers who are qualified to assess all facets of a manuscript. Further, the lack of consensus on the function of peer review makes it hard to evaluate success. The research community has not agreed on one common definition of peer review quality across disciplines. Measuring the quality of a peer review requires some standard to measure against, and no such standard exists. Various paradigms exist on how to study peer review, both experimental and nonexperimental ones. Collecting experimental data on peer review is especially difficult, given randomizing papers to different peer review practices (to measure outcomes downstream) could raise ethical concerns.

### Predatory Journals.

To add to the complexity of assessing the quality of peer review, the arguably perverse incentives of the academic ecosystem (citation) have enabled the emergence of “predatory journals”—that is, journals with lax or nonexistent peer review that publish articles in exchange for a fee. It has been estimated that there were around 8,000 predatory journals that “published” nearly half a million articles as of 2014 (24). A survey-based analysis of why authors publish in predatory journals (25) identified a lack of awareness of the reputation of the journals as a major factor. However, some scholars indicated that they would continue publishing in predatory journals if their institution recognized those publications, in line with the pressure to “publish or perish.” Finally, scholars in low- and middle-income countries (LMIC) expressed concerns that reputable Western journals might be prejudiced against non-Western authors and that they, therefore, felt more comfortable interacting with a non-Western journal, irrespective of its reputation (25). The quality of peer review is not improved by the emergence of journals for which the economic incentives are perfectly aligned with the number of articles that they publish; quality peer reviews do nothing else than decrease profits for these journals. Therefore, predatory journals make the situation worse and reduce the signal-to-noise ratio in the literature.

### Biases.

The impartiality of peer review may be jeopardized by individual and systemic biases that lead to favoring some authors, topics, methods, or groups and disfavoring others (e.g., overrepresentation of majority members reviewers, 26). For example, an author’s institutional affiliation may influence acceptance decisions considerably. Compared to double-blind review, single-blind review of medical conference abstracts favored authors from prestigious academic institutions in the United States and other English-speaking countries (27). Likewise, at an Association for Computing Machinery conference, reviewers who could see authors’ names and affiliations were significantly more likely than their double-blind reviewers to recommend accepting papers from famous authors, top universities, and top companies (28).

Because peer review is a system of multiple individuals interacting within institutional practices and field norms, small biases can accumulate into large disparities (29). For example, homophily between reviewers and authors (sharing gender, scientific lineage, or country of origin) is associated with more positive manuscript decisions (30, 31). Even in the (hypothetical) total absence of bias in individual-level decisions, group disparities emerge because the same decision can yield disparate impact given differential treatment, experience, or position at other points of the system.

Peer review biases are highly sensitive to context; social identity biases are apparent in some studies but not others. For example, the journal *Behavioural Ecology* transitioned from single-blind to double-blind review and saw a significant increase in the acceptance rate for manuscripts with women first authors (32), but one review of 145 journals found that women authors were favored when their identity was known to reviewers (26), while other studies suggest that there is no bias either for or against female authors (33–36).

### Poor Reliability.

Peer review can yield unpredictable and somewhat random outcomes. A meta-analysis of 45 different studies of the peer review process concluded that the average correlation between two reviewers’ ratings of the same submitted manuscripts was just 0.34 (37). Examinations of the way in which grant proposals are evaluated by outside experts have reached comparable conclusions (38, 39). In economics, medicine, and bioscience, there are many examples of prominent journals and funding agencies rejecting work that later became citation classics or led to Nobel Prizes (40–42). The reliability of peer reviews appears to be no higher in the natural sciences than in the social sciences or humanities and no higher in the 2000 s than in the 1970 s, 80 s, or 90 s (37). Although there are rare situations where different reviewers are asked to apply different criteria—for example, when a statistician is enlisted to comment on a submission’s statistical methods—studies of the reliability of peer review commonly focus on settings where reviewers are asked to rate articles on the same scale.

Disagreements in science are normal and can even be crucial to scientific progress when they lead to critical tests that decide between competing theories. However, such constructive disagreements typically take place in the open after competing theories and evidence have been published. Disagreements among peer reviewers before publication are not necessarily constructive and indeed raise questions about how much peer review improves authors’ work or ensures that only the best articles are accepted. The idea that peer review ensures the quality of published work is problematic if reviewers do not agree among themselves and cannot claim to represent the field’s consensus. In some cases, authors may be overfitting their work to a small and unrepresentative sample of potential readers, making changes that a wider readership may not value.

In the social sciences, additional reviewers are often recruited after an article has been revised and resubmitted. The ostensible purpose of second-round reviewers is to validate the quality of the revised paper, but given the low reliability of peer review, second-round reviewers often raise novel concerns, disagree with changes made in the first round, and make the final outcome less predictable. We think that the practice of inviting new reviews after revision may cause more trouble than it solves.

## Proposed Solutions

Is peer review dead, or “long live peer review?” Various solutions have been proposed to address the aforementioned challenges (Table 1). Here, we discuss the merits and limitations of some of the potential remedies for enhancing the integrity and fairness of the peer review process.

### Open and Transparent Peer Review.

Calls for transparency are increasing to mitigate the loss of credibility of peer review (43). How might transparency be managed?

The term “open peer review” comprises two distinct processes. The first, which we describe as “transparent review,” entails publishing peer review histories—editorial decision letters, reviewers “comments, and authors” responses. This approach has gained popularity but varies in its implementation. For instance, some journals like *Meta-Psychology* publish all elements of the peer review process as they occur. Others, like **Collabra: Psychology*,* only publish the peer review history of accepted manuscripts upon article publication.

The second type of open peer review involves “signed reviews”—i.e., sharing reviewers’ identities with authors. This can happen as a matter of policy (i.e., a journal could require that reviews be signed), or it could happen voluntarily by reviewers choosing to sign their reviews. Some journals have a policy that requires reviewers to always sign their reviews (e.g., *F1000Research*). Most journals leave it up to the reviewers’ decision. At the other extreme, many journals prohibit signed reviews and may even remove reviewers’ names if a reviewer signs their review.

Some journals (e.g., *Behavioral and Brain Sciences, Journal of the Royal Statistical Society*) invite comments and discussions to target articles and publish them together. These comments are akin to peer reviews as they provide scrutiny and context to the reader on the authors’ tenets. Sharing comments in this open format is rewarding for the reviewers as their contributions count as citable publications.

The value of signed reviews is debatable. Signed reviews may incentivize reviewers to write more effective assessments and avoid unprofessional content; they may facilitate the identification of conflicts of interest; and they may increase the journal's accountability when combined with transparent peer review by enabling more details of the peer review process to be studied. If all reviews were public and signed, many metaresearch questions could be studied, shedding light on networks and dynamics in peer review (e.g., are early career scholars more likely to write harsh or positive reviews? How evenly is the reviewer burden spread across career stages, geographical locations, etc.?).

The main argument against signed reviews is that reviewers may be less critical, particularly toward powerful researchers (and if signing is mandatory, they may be less willing to accept the review request in the first place). It is less clear whether reviewers are more likely to identify themselves when their evaluation is positive or whether forced signing produces more positive reviews (44). Fear of retaliation would likely have a greater impact on less-privileged reviewers (e.g., early career, minority, and scholars from less prestigious institutions). Concurrently, powerful researchers would amplify their privilege. Further, reviewers may sign their positive reviews when they wish to garner favor with the authors. This introduces external pressures and consequences to the peer review process that may undermine scientific integrity and progress.

### Increasing Peer Review Reliability and Validity.

To enhance the reliability and validity of the peer review system, academics could consider reviewer training, larger samples of reviewers, and reproducibility checklists.

It is likely that training is mostly happening informally—for example, when a senior researcher invites a student or junior colleague to prepare a review together. Currently, there are only a few systematic training resources, such as online training modules (45) and instructions provided to panelists reviewing grant proposals for funding agencies. While a substantial proportion of scientists indicate that they wish to receive training (46), there is not much evidence that such training is effective (47). Indeed, communicating the journal expectations had only a slight impact on the quality of reviews (47). Some practices that might increase reliability—for example, encouraging reviewers to guess whether other reviewers will like the paper—might have the undesirable effect of discouraging debate and encouraging intellectual monoculture. Even if training were effective, offering it on a large scale would be challenging with some circumscribed exceptions (e.g., focused training to a pool of compensated reviewers visiting a federal agency for a two-day stint on a review committee).

To increase reliability, journals could recruit a larger sample of reviewers and average ratings across them. But this option seems costly, given editors are already having difficulty recruiting reviewers and getting reviews completed in a timely fashion. Moreover, increasing the number of reviewers may make achieving consensus even more elusive.

Some journals (and leading AI conferences) have added reproducibility checklists as part of the paper submission process, which aims to increase the reliability of research findings, including items related to experimental design, statistical model evaluation, and model calibration (48). Although checklists seem to enhance code and data exchange, their effect has not been extensively assessed (48). Some conferences in computer science, such as IEEE Supercomputing, have a volunteer reproducibility project that evaluates submitted papers for repeatable results and awards badges to successful replications. However, this strategy may not be effective in other areas. Randomized studies in medicine revealed no effect in reminding reviewers to apply reporting guidelines and checklists to the quality of their review (49).

### Preprint Peer Review.

Preprints have become a popular method for disseminating scientific work in diverse fields. The physical sciences championed this approach: ArXiv includes over 2 million documents as of September 25, 2023. Preprints are also popular in the social sciences (mainly economics), especially Social Sciences Research Network (with over a million and EconStor with over two hundred thousand documents. Uptake has been slower in biomedicine, but the COVID-19 pandemic provided some impetus that is reflected in the numbers for medRxiv (n = 46,158) and bioRxiv (n = 208,226). By 2020, there were 57 preprint servers reviewed in ref. 50.

Reprints allow for rapid dissemination of research. Peer review time varies across academic disciplines. For example, it is significantly slower in economics than in the natural sciences (51). In theory, a slower process could result in a better, more thoughtful outcome, and yet, in practice, delays are often unrelated to greater scholarly attention (52, 53). Reprints provide a solution. Indeed, to address the slow publication process, economists rely on (and cite) working papers in ongoing scientific work and promotion decisions, hence bypassing the peer review.

The potential pros of preprint servers further include the possibility for immediate feedback and reduction of publication bias. However, these advantages are not guaranteed, as many preprints do not attract attention or feedback (54). Despite this major caveat, preprints can make scientific information more readily available. Moreover, preprints are available to the entire scientific community rather than only a few journal peer reviewers. The challenge is how to engage competent reviewers in assessing preprints and in making a difference through proposed revisions that are adopted. Another possibility is to use AI approaches that can scale up massive reviews of preprints. AI has the potential to assist experts in otherwise tedious tasks, such as analytical checks, code checks, and grammar checks. However, the accuracy and utility of such approaches need careful validation (55). AI tools may perform suboptimally in the absence of expert human guidance.

There are also several concerns surrounding the advent of preprints. By definition, preprints have not been peer-reviewed when posted, so they miss whatever value peer review has. In the extreme case, if preprints contain serious errors or are posted by bad-faith actors, they may contribute to the dissemination of false information. Misinformation may be particularly problematic for topics that have implications for public health (56). These concerns generate debate as to whether the gatekeeping function of preprint platforms should be strengthened. However, genuine strengthening would require far more resources and would contradict the main advantage of preprints, which is to make information available speedily before it has been vetted.

Both the pros and the cons of preprints may be currently exaggerated. Empirical studies that have assessed papers at the preprint stage versus the final publication typically show only minor changes (57, 58). These changes may reflect the impact of both preprint peer review and journal peer review (if preprints are posted before journal peer review), and they suggest that review makes little difference. One promising solution is represented by Peer Community In^*^ (PCI) initiative, which offers peer review and recommendations for preprints. Over 130 PCI-friendly journals already accept these recommendations and can offer publication in their outlet, often without further peer review. It propagates the idea that peer review can be organized by the research community outside of the journal system, which should come with a number of advantages, such as saving the time it takes to move from journal to journal with submissions.

### Reviewer Incentives, Recognition, and Availability.

Finding reviewers has become more challenging (59). According to Publons, the number of reviewer invitations to secure a single reviewer rose from 1.9 in 2013 to 2.4 in 2018 (60). The main reasons are likely the absence of an incentive and the declining ratio of qualified reviewers to the number of papers needing reviewers (61). In most cases, reviewing does not lead to monetary compensation or advance a reviewer’s career but is done for social reasons (e.g., 41% of researchers in the Publons survey gave “reviewing is part of my job” as a reason). That incentive may no longer be enough.

Monetary compensation of reviewers has been suggested in view of the very large profits earned by some corporate publishers (62), earned from academics’ free labor in both authoring and reviewing (63). Assessing the allocation of public or private funds toward reviewer compensation is a debate worth having, but many journals—especially those that are not published by large corporations or wealthy universities—lack the money to compensate reviewers, and reviewers are often compensated with only token payments that do not meaningfully change their incentives to accept reviews or conduct them carefully.

Another prominent problem is that many valid points in the reviews never make their way to the papers, leaving reviewers feeling unsatisfied and depriving readers of important information. To remedy this, a number of journals have begun publishing peer reviews in recent years (e.g., *Meta-psychology, eLife, Advances in Methods and Practices in Psychological Science*, *BMJ One* and *BMC* journals, as well as some conferences, e.g., NeurIPS and ICLR). More journals could follow this practice. Encouraging this model, along with objective verification of reviewer status and number of reviews, through platforms such as Publons could lead to increased incentives for reviewing. In addition, some services, such as Reviewer Credit (reviewercredits.com), offer benefits such as statistics consulting and English editing services in exchange for accumulating reviewing points.

As mentioned above, publishing peer reviews would also have other benefits, such as allowing stakeholders to compare the quality of reviews in different journals and uncovering more constructive review practices. Publishing of nonanonymized peer reviews does have its problems, however. For example, it can lead to writing less critical reviews, although this has not been empirically validated (64).

### Improving Recruitment of Reviewers.

Reaching out to a broader and more diverse pool of potential reviewers is a potential remedy for the shortage of reviewers. For example, many countries, especially in the Global South, supply a disproportionate number of manuscripts relative to how many they review (60). This would require that these candidate reviewers be more easily found and that they are motivated to perform the reviews.

Traditional recruitment of reviewers occurs via a direct approach (usually email). Another route is broader recruitment or “crowd-sourcing.” PREreview^†^ is an example of such initiatives that recruit pools of reviewers to engage with pools of openly available manuscripts such as preprints. In addition, some journals (such as *Meta-psychology*) announce when a preprint is under review and invite anyone interested to comment on it. While such initiatives remain relatively small-scale, most did not exist 10 y ago and may continue to grow. At this point, we acknowledge that this and other examples of reform initiatives may not stand the test of time; we believe that they represent significant efforts to improve the system.

To address editors’ limited knowledge and recall, several journal management systems now provide automated recommendations of candidate reviewers with algorithms that may include machine learning (65). This has the potential to broaden the pool of reviewers used by journals and reduce the number of reviewer invitations required. Although some models seem to outperform traditional recommendation techniques (65), the validity and relevance of the automated recommendations cannot be taken for granted.

Finally, the expectation that a reviewer will evaluate an entire manuscript seems increasingly burdensome as science advances, with the associated increase in specialization as well as interdisciplinary work. Asking reviewers to judge only the facets of a manuscript within their expertise may improve the reviewer response rate. This can be systematized in areas where articles are expected to follow certain guidelines or checklists, with different sets of specialists looking at different aspects of whether such criteria have been met.

Despite the low reliability and disagreements that are characteristic of peer review, editors often pass reviews to the author without comment, leaving authors to resolve the contradictions and idiosyncrasies. Instead, it would be more efficient and productive for editors to emphasize points where reviewers agree with each other or with the editor and to highlight suggestions made in a reviewer’s area of expertise (e.g., statistical advice given by a statistician). Other suggestions can be left to the authors’ discretion. In this way, the research can be revised more quickly and with more confidence that the revisions will satisfy a broader readership and not just a single reviewer.

### Transparent Editorial Process.

Should an editor take the importance of a paper into account when making a decision, or only the technical accuracy? And what dimensions of importance should play a role, and should the answer depend on the “status” of the journal?

Calls for transparency in peer review are frequently accompanied by calls for transparency in editorial decision-making. Existing models of transparency span a range of options, from providing detailed statistics about editorial decisions and time lags to publishing all editorial correspondence after a paper has been published. *Frontiers* journals employ an interactive portal where reviewers and authors directly engage in conversations during the publication process. Similarly, many machine learning conferences, such as the Conference on Neural Information Processing Systems use the OpenReview platform for their reviewing and editorial processes. Accepted articles are accompanied by their reviews and final judgment. While publishing editorial decisions increases transparency, most decision letters are not made public, meaning that the reasons underlying most editorial decisions are opaque. Most journals reject a large portion of papers submitted to them without reviewing them (“desk” rejections) as these papers are viewed as not relevant for the journal or judged to be not competitive enough to bother reviewers. Desk rejections may necessitate some thinking about how to standardize the process and make it fair (66).

To date, there has been remarkably little public or scientific attention devoted to the actual process of editorial decision-making. Tran et al. (67) analyzed the review process of a machine learning conference for all submitted papers. There, the review process is double-blind, but the authors’ names of both accepted and rejected papers are published after the review. As part of their analysis, Tran et al. show that review scores are more reproducible than the decisions made by the area chairs who make the final accept/reject decision. Hence, they argue adding reviewers will not necessarily reduce the randomness of decisions. A better option is to improve the decision process made by area chairs. Acceptance decisions are affected by whether papers have been published at *Arxiv* before the review process starts, which is allowed by most top AI and machine learning conferences. Top institutions seem to benefit most from this.

Anecdotally, the ways in which editorial decisions are made seem to fall into two broad categories: On the one hand, some editors primarily seek to integrate the opinions of reviewers into a decision that reflects the tenor of the reviews. Action letters tend to be short and refer to the reviews. This style is pervasive among journals in geophysics and computer science. One of the few scholarly papers discussing editorial decisions (68) leans toward endorsing this approach. The benefit of this approach is that editors shift control to reviewers they choose as subject matter experts, albeit at the cost of reducing editorial involvement in the publication decision. Consequently, editorial responsibility shifts to a moderator role where editors select appropriate reviewers in charge of journal quality. The impact of the editors is in desk rejection and in difficult situations where reviewers disagree. Disciplines such as cognitive science, philosophy, and psychology are a mixed bag. Even within the same journal, action editors differ substantially in how they arrive at their editorial decisions.

The reason for such variability in editorial styles is primarily the action editors’ wish for autonomy and flexibility. Such flexibility is undoubtedly beneficial, yet imposing some form of structure could add objectivity. Research in fields that rely on logical reasoning can—and must—be evaluated within a logical structure. Such a structure would have a hierarchy for assessment; it starts at higher levels with key questions like “Is the logic of the design sound for answering the research question?” the answer to which can be grounds for rejection and moves down to less critical issues such as adequate sample size, which can be addressed with a major revision, to yet more minor issues, such as discussing additional studies in relation to the reported results.

This hierarchy is important because addressing the lower-level issues is moot unless the higher-level issues are fixable/fixed. Unfortunately, many reviewers overlook the higher-level assessments in favor of commenting on less important details. When editors simply send all the reviews to the authors without providing any attempt at giving structure to the reviews, a long and cumbersome review process ensues, which may result in rejection after multiple rounds.

In editorial circles, the lack of success in reviewer training is often taken as evidence that any attempt to regulate the review process by imposing objective criteria is pointless. This conclusion may be premature. While reviewers’ training may not be effective, providing such training and structure for editors, who presumably start with most of that knowledge already in place, could bring more objectivity to the review process. The main objective of such a structure would be to demand that all editors at least assess the reviews within the hierarchical logical structure discussed above and provide a summary of the critical points in order of their severity. This system solves several problems brought up in various points in the current paper: a) It helps reconcile contradicting reviews. An issue must be addressed if it falls within the higher levels of the logical assessment hierarchy, even if only one reviewer deems it to be a problem. At lower levels, editors can advise an appropriate course of action. b) It can make waiting times shorter. For example, if existing reviews highlight fundamental logical flaws that cannot be easily addressed in a revision, the editor can make a case for not delaying an inevitable outcome by waiting for a late review. c) It can make the revision trajectory clearer. Once the critical problems are highlighted in the editorial summary in order of importance, it is easier to assess the likelihood of success for a revision. The editors can state from the start that the persistence of higher-level logical problems after the first revision is grounds for rejection. and d) It provides an abstract but objective, uniform backbone for editorial decisions across papers.

Some potential drawbacks of the above approach are also worth mentioning. i) It requires editors who have the ability to apply the hierarchical logical assessments to reviews. ii) It creates more work for editors. and iii) Not all aspects of an editorial decision may fit the hierarchical logical assessment outlined above. For example, the impact may be more subjective. It can be argued that item i) should be a given. Item and ii) can be addressed by splitting one position between two or more action editors, a practice that is becoming more popular. This is tied to the issue of editorial compensation, which must be addressed with publishers. Profit margins are high for most major publishers (69, 70). Editorial teams are in a good position to negotiate compensation, and such negotiations will be an important part of any efforts to improve the review process by distributing the editorial positions more widely and equitably between editors. Item iii) is where editorial discretion and flexibility may be practiced. The goal is not to box in the editors but to complement individual judgments with a semistructured framework to enhance transparency and uniformity.

Whether or not this approach will significantly improve the quality of the review process is an empirical question. But considering the possibility will open up discussions on the nature of the most appropriate hierarchical logical structure and will allow for conducting systematic studies on how implementing such a structure may improve the reliability of editorial decisions, as well as authors’ satisfaction with the timeline and quality of peer review.

### Empirical Testing and Randomized Trials.

Should the practice of peer review be subject to scientific testing? Empirical testing of peer review practices may use various study designs. Nonexperimental designs (e.g., comparisons of journals that use different peer review practices) offer observatory-type information and may suffer from confounds. Quasiexperimental designs, for example, interrupted time series with pre–post assessments when a peer review practice is changed, are more robust but not confound-free. Scientific publishing is a volatile environment, and many things may change over time, blurring the impact of any specific intervention.

The most rigorous designs, randomized controlled trials, are also the most demanding. They require journals to randomize the submissions they receive to different peer review practices; or multiple journals to agree to be randomized to different peer review practices. Proper randomization (largely) removes confounding biases. However, getting informed consent and participation is challenging; the randomized submissions or journals may constitute sets that are not representative of the whole publishing milieu; and experimental circumstances may deviate from the real world (in particular when authors/reviewers/editors know they participate in experiments). Moreover, trials meet with resistance in some scientific circles that are unfriendly to auditing. Finally, a peer review intervention that works in one or a few explicitly investigated journals may have unintended or downstream effects in other journals. For instance, if an intervention aims to make reviewers and editors ask more routinely for the raw data to be disclosed, articles that disclose data will grow considerably if a publication demands data sharing, which could influence broader scientific sharing practices and policies. Perhaps authors who want to share submit more work to that journal, but sharing decreases in other journals without any net improvement overall.

Despite these caveats, many randomized trials have been performed on peer review practices. A recent systematic review and network meta-analysis identified 24 randomized trials (71), including eight on the effect of interventions at the author-level, 16 at the reviewer-level, and 3 at the editor-level (3 studies investigated interventions at multiple levels). Reviewer-level interventions improved quality and (except for blinding) increased the duration of the review process, but the effects were small. Moreover, this may apply only to the few interventions assessed and the specific settings/journals and may not be generalizable. Author- and editor-level interventions had similar point estimates, but the effects carried considerable uncertainty and were not nominally significant—perhaps because of more limited data. Another systematic review (64) of 22 randomized trials concluded that the largest improvement in manuscript quality (e.g., clarity of the empirical report) was seen with the addition of a statistical reviewer (72, 73). It also found that some interventions affected the rejection rate, in particular, open review markedly reduced the rejection rate. A third systematic review (74) found that blinded review may partly mitigate gender bias. Clearly, far more randomized trials are needed, and they need to test a wider spectrum of interventions. The same applies to the peer review of other scientific work, for example, grant proposals, where the evidentiary base is even thinner.

A welcome development is the generation of consortia and groups of journals that are interested in participating in journalology studies, including experimental ones where peer review and other research practices can be tested (75). Multicenter (multijournal) trials may be more generalizable and less likely to be affected by biases; thus, they are also more likely to have “negative” results for specific peer review interventions (49).

### *Anything Goes?* Why Uphold Peer Review at All.

Peer review is a modern invention. Although independent referees were solicited as early as 1832 by the Royal Society, science was conducted successfully largely without the formal implementation of peer review until the 1970 s. Crick and Watson’s 1953 Double Helix paper was unvetted. Einstein’s *Annus mirabilis* papers were published in *Annalen der Physik* in 1905 without any peer review. Doubts concerning the effectiveness of peer review, and whether it may be worth abolishing^‡^, should be entertained rather than outright dismissed.

Two arguments for the abandonment of peer review are worth emphasizing (76). First is the **'argument from history*'*. Peer review would have prevented the publication of some major scientific discoveries in history. Galileo’s conception of the universe was controversial at the time. Exonerated in hindsight, Galileo relied heavily on rhetorical methods in addition to scientific observation (77), and his empirical investigations did not hold up to scrutiny by scientific standards either. The case of Galileo highlights a profound consequence of peer-based authority, particularly in publications with a high rejection rate: the creation of intellectual monoculture. Peer review judges the validity of a study and an article’s claims against the backdrop of currently validated knowledge and measures. However, most breakthroughs we see as paradigmatic to science were routinely achieved *in spite,* not because of consensus. Examples of ground-breaking discoveries that were not driven by the prevailing consensus at the time include those of Barbara McClintock (78), Katalin Kariko (79), or Hans Krebs (80).

Second is *the ''*argument from pluralism*'*. Scientific pluralism holds that research advances best when it uses multiple, sometimes contradictory models and methodologies (81). Take the Global Positioning System (82): GPS uses satellites locked in place by Newtonian physics and an atomic clock regulated by quantum mechanics. It is adjusted by special and general relativity to map the round planet on a geostatic grid in order to advise humans on the ground from a flat earth perspective. A reviewer may easily judge this incoherent. Yet, it works. False models can act as a means to truer theories (83, 84), so much so that failures drive science as much as its successes—if not more (85, 86).

Objections to the abandonment of peer review largely involve the *social* status and impact of science more than concern about scientific discovery (87). One concern is distributing misleading or fudged studies without peer review. Science has a reputation to uphold. But given the apparent flaws of bogus studies being published despite passing peer review, the effectiveness of peer review must be called into question. Meanwhile, many scholars already read unreviewed manuscripts on preprint servers to keep up with a field’s advances, while the general public is given a false sense of security by trusting published findings due to expert review (88).

The devaluing of “quality research” is another concern for some researchers if people can post whatever they want as research. Nonetheless, the lack of professional competition to jump the review hurdles to publish in prestigious venues may reduce incentives for “low risk, high (social and media) gain” research, and even fraud. Abandoning peer review could also help address an often-neglected geographical imbalance: Prestige bias and slow reviewing commonly prevents scholars in low- and middle-income countries from participating in “global science.” LMIC research is less influential globally due to the lack of prepublication data and lower academic publication rates. Due to delayed publishing rates and reinforcing invisibility, LMIC scientists are reluctant to discuss prepublication data with nonclose contacts (89). Peer review can turn a world of science into a remote island.

Fear of publication anarchy due to “peer-freed” research might be less detrimental to science than a bottleneck monoculture. Still, this proposal will sound too radical to many academics. As perspectives differ sharply, including among the authors of this paper, this disagreement might lead to different methods and different ideas that could yield viable alternatives. The possibility of abandoning peer review thus ought to be considered for the ideal of peer review to be re-envisaged.

A number of factors may affect peer review in the near and mid-term future (Table 2). They include changes in technology, reward systems, publication practices, peer reviewer fatigue, and the influence of diverse stakeholders external to science, e.g., media, social media, and infodemics. The impact of these factors is unpredictable, and there is potential interaction among them.

**Table 2.** Evolving factors that may affect peer-review in the near and mid-term future

Advent of AI and large language models
Expanding scientific workforce
Reward systems in research and academia
Diversification of publishing modes
Advent of mega-journals
Predatory and unethical practices (e.g., paper mills, citation cartels)
Peer-reviewer fatigue
New standards, methods, and influence of postpublication review
Interface between science and society, review in media, social media, and infodemics

## Conclusions

Peer review works when it improves research and its presentation. To improve peer review, scientists must resolve the challenges we collectively examined in this paper. Noticeably, the presentation of these challenges draws us into various somewhat related yet disparate aspects of science as an epistemic practice and science as a growing social or institutional community. While many problems with peer review listed here are not problems exclusive to science, we seem to hold science to higher standards. Science has managed to secure a privileged position within modern societies (90), and thus, we are struggling to see that our expectations and practices of peer review are aligned with this exalted position.

Ultimately, we may need to revisit a fundamental question: What is peer review for, and for whom? Accordingly, besides the need for more empirical evidence, the underlying problem also has philosophical dimensions. Efforts to tackle the challenges of peer review with experimental approaches and data collection, including the strategies proposed in this paper, must be coupled with clearly defined objectives and purposes and accompanied by conceptual analysis. The philosophical kernel of peer review is how we connect its value and challenges with our image of science. We need to be clearer on what we want science to be(come) in order to guide decisions about the future of peer review.

## Data Availability

There are no data underlying this work.

## Footnotes

^*^https://peercommunityin.org/.

^†^https://prereview.org/.

^‡^https://www.experimental-history.com/p/the-rise-and-fall-of-peerreview.
